# Parliament and the Professions

**Published:** 1920-08-14

**Authors:** 


### Parliament and the Professions.

### Poor-Law Institutions.

ANSWERING a question as to the effect of old-age pensions upon workhouse accommodation, the Minister of Pensions stated that there is at present a margin of accommodation in Poor-Law institutions due to some extent to the cause mentioned. A large amount of the vacant accommodation was used for other purposes during the war, and whenever possible arrangements for setting free such institutions for other uses are encouraged.

### Medical Examinations of Pensioners.

IN answer to a question addressed to the Minister of Pensions, it was stated that from July 1, 1919, to June 30, 1920, Medical Boards have carried out 1,225,567 medical examinations. So far as possible men are given the opportunity of being boarded in their own towns.

